# Parkinson-Related Changes of Activation in Visuomotor Brain Regions during Perceived Forward Self-Motion

**DOI:** 10.1371/journal.pone.0095861

**Published:** 2014-04-22

**Authors:** Anouk van der Hoorn, Remco J. Renken, Klaus L. Leenders, Bauke M. de Jong

**Affiliations:** 1 Department of Neurology, University Medical Center Groningen, University of Groningen, Groningen, The Netherlands; 2 Neuroimaging Center, University Medical Center Groningen, University of Groningen, Groningen, The Netherlands; Federal University of Rio de Janeiro, Brazil

## Abstract

Radial expanding optic flow is a visual consequence of forward locomotion. Presented on screen, it generates illusionary forward self-motion, pointing at a close vision-gait interrelation. As particularly parkinsonian gait is vulnerable to external stimuli, effects of optic flow on motor-related cerebral circuitry were explored with functional magnetic resonance imaging in healthy controls (HC) and patients with Parkinson’s disease (PD). Fifteen HC and 22 PD patients, of which 7 experienced freezing of gait (FOG), watched wide-field flow, interruptions by narrowing or deceleration and equivalent control conditions with static dots. Statistical parametric mapping revealed that wide-field flow interruption evoked activation of the (pre-)supplementary motor area (SMA) in HC, which was decreased in PD. During wide-field flow, dorsal occipito-parietal activations were reduced in PD relative to HC, with stronger functional connectivity between right visual motion area V5, pre-SMA and cerebellum (in PD without FOG). Non-specific ‘changes’ in stimulus patterns activated dorsolateral fronto-parietal regions and the fusiform gyrus. This attention-associated network was stronger activated in HC than in PD. PD patients thus appeared compromised in recruiting medial frontal regions facilitating internally generated virtual locomotion when visual motion support falls away. Reduced dorsal visual and parietal activations during wide-field optic flow in PD were explained by impaired feedforward visual and visuomotor processing within a magnocellular (visual motion) functional chain. Compensation of impaired feedforward processing by distant fronto-cerebellar circuitry in PD is consistent with motor responses to visual motion stimuli being either too strong or too weak. The ‘change’-related activations pointed at covert (stimulus-driven) attention.

## Introduction

Goal-directed movement implies sensorimotor transformations that enable effective interactions with surrounding space and objects placed in it [1]–[4]. Walking is a specific motor action characterized by multi-limb movements organized to achieve fluent locomotion, which heavily relies on visual representations of near and distant spatial environment. Aside from putative targets and obstacles that may guide motor control, forward locomotion itself generates a characteristic streaming motion of environmental features through the visual field. This ‘optic flow’ implies a radial expansion from the central point on the horizon ahead, provided that the observer does not fixate a specific object [5]. Optic flow is not only a visual consequence of locomotion, it also supports gait [5] and generates the illusion of forward self-motion when presented on a static display [6]. This visual motion stimulus thus appears to be intimately connected with the cerebral organization underlying locomotion. Support for the latter might be inferred from the involvement of premotor activations in recent functional Magnetic Resonance Imaging (fMRI) studies [7]–[9]. In the present study, we used fMRI to explore responses of visuomotor circuitry evoked by visual motion stimuli in elder healthy subjects and patients suffering from Parkinson’s disease (PD).

In PD, the effect of external stimuli on movements, either in a supporting or obstructing fashion, appears to be even stronger than in healthy subjects [10]–[13]. This also holds for walking [14], [15]. In this respect, the effect of optic flow is underscored by the observation that improvement of gait by presentation of parallel traverse lines on the walking surface [7]–[9] is dependent on the perceived motion flow of stripes due to walking itself [8]. This was assessed using stroboscopic light indicating that responsiveness of involved visuomotor circuitry favors dynamic more than static environmental features. The latter is consistent with the supporting effects of optic flow presented on screen during treadmill gait in PD [14], [16], [17] and visual motion manipulation during ground walking [14], [16], [17].

The inability to maintain the cyclic pattern of walking is reflected in freezing of gait (FOG), which is a most disabling symptom that frequently occurs in PD patients [18]. A provocative environmental factor is e.g. the transition to narrow spaces like a corridor or doorway [19]–[21]. On the other hand, visual stimuli may also provide support to overcome FOG [7], [14], [17], [22], [23]. Given the indications that optic flow provides a basic cue for gait support, we recently employed a treadmill paradigm with presentation of a wide-field radial flow pattern that gradually narrowed, mimicking the illusion of entering a narrow corridor [24]. During the transition to a narrow flow field, backward displacement due to slowing of gait was seen in PD patients particularly with left-sided symptom dominance.

In attempts to explain the failure to maintain performing a visuomotor action in circumstances of incongruence between visual stimuli and intended movement, disequilibrium between medial and lateral premotor functions should be considered. Visual information contributing to motor control is particularly processed along a dorsal (occipito-parietal) pathway involving lateral premotor regions, while internally driven action is particularly funnelled via the (pre-) supplementary motor area (pre-SMA and SMA), located on the medial hemisphere surface [11], [25]–[29]. These medial premotor areas are dominant output targets from basal ganglia-thalamic circuitry [30], [31] and indeed functionally affected in PD [32]–[34]. As a consequence, adverse external stimuli or loss of supporting stimuli cannot be met by internally driven action initiation mediated by SMA activity. In PD, reduction of optic flow stimuli at approaching a narrow doorway may thus result in FOG because a compensatory motor drive cannot be recruited due to impaired SMA function. In young healthy subjects, we indeed gained support for this concept by demonstrating that the transition from a wide to narrow optic flow field evokes (pre-)SMA activation [35].

In line with these arguments, we aimed to test the hypothesis that observing the transition from a wide to narrow optic flow field also recruits medial frontal (pre-)SMA activation in elderly healthy subjects while it is reduced in PD patients. Moreover, as wide-field optic flow has a stronger influence on gait in PD than in healthy subjects, we wanted to assess whether this condition might evoke a stronger effect on the dorsal premotor cortex in the patients. As optic flow represents a basic visual motion stimulus, intrinsically linked to locomotion, manipulation of this specific stimulus pattern was considered to provide a tool to explore PD-related changes in the dorsal visuomotor pathway.

## Methods

### Ethical Statement

The study was approved by the Medical Ethics Committee of the University Medical Center Groningen. All subjects gave written informed consent according to the declaration of Helsinki. Procedures and task instructions were explained at least one week before scanning as well as immediately before the experiment.

### Subject Inclusion

Initially, 24 PD patients were tested, of which 7 suffered from FOG (PD_FOG). For these 7 FOG patients (mean age 62.1 years, SD±9.5; three males), FOG was documented by the FOG questionnaire translated in Dutch [36]. Two PD patients without FOG (PD_nFOG) were excluded from further analysis because they exhibited too many movements during scanning, thus resulting in the final inclusion of 15 PD_nFOG patients (60.9 years±12.1; nine males). In addition, 15 age-matched elderly healthy controls (HC) (60.5 years±6.2; nine males) were included (Table 1). The conclusions we finally draw in this study are based on the dataset of these two balanced groups of 15 subjects each, which was optimally constituted for rigorous image statistical analysis. None of the subjects had neurological, ophthalmologic or lower extremity disorders, other than PD in the patient group. Patients were tested after twelve-hour medication withdrawal to obtain optimal insight in PD-associated dysfunction.

**Table 1:** Subject characteristics.

	PD			HC
	PD total	PD_nFOG	PD_FOG	
**Number (Males/Females)**	22 (12/10)	15 (9/6)	7 (3/4)	15 (9/6)
**Age (yr)**	61.3 (11.2)	60.9 (12.1)	62.1 (9.5)	60.5 (6.2)
**MMSE (0–29)**	27 (26–28)	27 (26–28)	27 (27–29)	27 (26–28)
**Education level (1–7)**	6 (5–6)	5 (5–6)	6 (5–6)	6 (5–6)
**PD duration (yr)**	7.1 (4.2)	6.1 (3.8)	9.4 (4.2)	na
**L-dopa equivalent dose (mg)**	690 (367)	609 (276)	871 (490)	na
**Modified Hoehn & Yahr (1–5)**	2.5 (2.0–2.5)	2.5 (2.0–2.5)	2.5 (2.0–2.5)	na
**UPDRS III (0–56)**	25.1 (8.3)	21.6 (4.9)	32.7 (9.3)	na
**FOG questionnaire (0–24)**	4.1 (4.8)	1.2 (1.3)	10.1 (3.7)	na
**Dominant PD side (R/L)**	13/9	8/7	5/2	na
**Block handling time (s)**	4.8 (2.1)	5.2 (2.3)	4.1 (1.7)	3.8 (1.1)
**WAIS block design T-value (0–100)**	47.4 (12.4)	44.6 (12.0)	53.4 (12.0)	50.2 (8.0)

Subject characteristics quantified by mean value (standard deviation) or by median (interquartile range) for non-Gaussian data. Possible range of variables are displayed behind the variable names. Range of the Education scale is explained in the methods. The MMSE sum score did not reach 30 because the maximum score on the item ‘orientation in place’ was 4 and not 5. The local convention is due to the fact that province (state) and city are both named Groningen, which is the reason not to ask for the city name after the name of the state has been given. Abbreviations: FOG = Freezing of gait; HC = healthy controls; L-dopa = Levodopa; MMSE = Mini mental state examination; PD = Parkinson’s disease; PD_FOG = PD patients with FOG; PD_nFOG = PD patients without FOG; R = right; L = left; UPDRS = unified Parkinson’s disease rating scale; WAIS = Wechsler adult intelligence scale; na = not applicable; mg = milligram; s = seconds; yr = years.

### Behavioral Assessments

Mini Mental State Examination (MMSE) [37] and the Edinburgh handedness inventory [38] were conducted to underscore that participating subjects were non-demented and right-handed respectively. Education level was classified with a Dutch education scale, ranging from 1 (uncompleted elementary school) to 7 (university degree) [39]. In patients, the motor part of the Unified Parkinson’s Disease Rating Scale (UPDRS) [40] was conducted immediately prior to the experiment. Furthermore, data concerning the modified Hoehn and Yahr scale [41], levodopa-equivalent dose, disease duration and dominant side of PD symptoms were collected. Disease duration and dominant side of PD symptoms were derived from the clinical file. The side of symptom dominance, which was consistent with the UPDRS asymmetry, was similar to the side of symptom onset.

The block design subtest of the Wechsler adult intelligence scale (WAIS) III [42], representing visuomotor transformation, was applied as general indicator of right hemisphere function. Scores were obtained in the standard manner with the exception that subjects were not restricted by time limits, although the standard time limits were used as cut-off for the raw test scores. Rough scores were converted to scaled scores that provided the percentile values using norm tables for the subject’s corresponding age. From these percentiles a commutation to T-values was made. As reduced movement speed in PD might be a confounding factor in the block design test, general ‘block handling speed’ was assessed, twice before and twice after performing the block design test. To that end, a series of four blocks was turned around as quickly as possible, in such a way that the side facing the table was turned upwards. The four blocks were subsequently arranged in a square without considering a specific pattern.

Wide-field visual presentation of a radially expanding flow of dots (optic flow) generates the illusion of forward self-motion [6]. Immediately following the fMRI scanning protocol that included the presentation of visual motion conditions, subjects were asked to rate this illusion for the conditions wide-field flow (FW), narrow-field flow (FN), wide-field static dots (SW) and narrow-field static dots (SN) on a scale from zero to maximal (0–10). All behavioral test were performed on the same day as the fMRI experiment.

### Statistical Analyses of Subject Characteristics and Performance

Statistical tests of group characteristics, PD parameters and behavioral assessment of the WAIS block design test were conducted with SPSS 19 (SPSS Inc: Chicago, IL, USA). Normality of data was tested with the Shapiro-Wilk test. Differences between groups for normal distributed data were tested with an independent t-test or one-way ANOVA for respectively two or more groups. If equal variance was violated according to Levene’s test for equality of variances, we used the adjusted values of the t-test. Non-Gaussian data were tested with a Mann-Withney-U test or Kruskall Wallis for respectively two or more groups. For the latter, follow-up pairwise comparisons were conducted using a Wilcoxon test and correcting across these comparisons using the least significant difference procedure.

With regard to the ratings of illusionary perceived forward self-motion, differences between the HC, PD_nFOG and PD_FOG were tested with the Kruskal-Wallis test for non-Gaussian data (*p*<0.05, two-tailed). Within group differences were tested with the Friedman test for non-Gaussian data (*p*<0.05, two-tailed). Follow-up pairwise comparisons were conducted using a Wilcoxon test and correcting across these comparisons using the least significant difference procedure.

### Task Design fMRI

During scanning, subjects watched stimulus patterns on a screen via a mirror placed approximately 11 cm from the face, with a mirror-screen distance of 64 cm. The screen dimensions were 44 by 34 cm, comprising 25.5° by 32.7° of the visual field. A projector with a monitor refresh rate of 30 Hz and a resolution of 1024×768 pixels (Barco, Belgium) projected the computer-generated stimuli on the screen. The experimental conditions were presented using the “Presentation” program (Neurobehavioral Systems, Inc. Albany, CA, USA).

All conditions consisted of white dots on a black background in the lower half of the screen, with a mean density of approximately 340 dots. A small horizontal line was used for gaze fixation and placed at the middle of the virtual horizon in all conditions. Dot patterns were presented either in static or in dynamic mode. In the latter, stimuli consisted of 25 frames per second. Each dot radially moved with increasing speed from the center towards the bottom edge of the screen in two seconds. The mean speeds of a dot was 4.9° in the first second and 7.9° in the last second, reaching a maximum of 26° per second at disappearance from the screen. Dots proportionally enlarged along their trajectory. This dynamic stimulus pattern of wide-field flow (FW) generated the illusionary perception of forward self-motion.

Ordered in two conditions, FW was interrupted either by narrowing the flow field or by deceleration of dot movement. The gradual transition to a narrow flow field (FtN) was established by dark gray surfaces that expanded from the horizon in both upward and downward direction in 1.8 seconds, leaving a midline vertical narrow flow field (FN) of 1.3°, 4 percent. This transition evoked the natural illusion of entering a narrow corridor like a doorway. Stopping wide-field flow (FtS) was achieved by a linear reduction of dot velocity within 1.8 seconds, leaving a stationary wide field. In order to assess specificity of interrupting particularly wide-field flow, transition (StN) from a stationary wide field (SW) to a stationary narrow field (SN) with equal surfaces as in FtN served as control. After each of the transitions, the stimulus pattern lasted for 2.2 seconds. Until onset of the next stimulus condition, a rest condition with the central fixation line on an empty black background was shown, supporting continuous gaze fixation.

Stimuli were presented in a block design with six blocks ordered in two runs. In between the runs, a T1 anatomical scan was made. A block was constituted by six repetitions of each of the three stimulus trials [FW-FtN-FN], [FW-FtS-SW] and [SW-StN-SN], respectively. Trials were randomized for each block. In order to avoid stimulus anticipation, the duration of a stimulus pattern before transition varied between 5 and 10 seconds (in whole numbers). The intervening rest condition with only the central fixation bar lasted 2–10 seconds. This design resulted in 36 repeats for each condition, except for FW with twice as much repeats.

### MRI Data Acquisition

Data acquisition was performed using a 3T Philips MR system (Philips Medical Systems, Best, The Netherlands) with a standard 8-channel head coil. A 3D T1-weighted anatomical scan was acquired with repetition time 9 ms, echo time 3.6 ms, flip angle 8°, field of view 232×256×170 mm, 170 slices without slice gap, voxel size 0.9×1×1 mm. Functional images were acquired with a gradient-echo T2* blood oxygen level dependent (BOLD) contrast technique using with a repetition time 2000 ms, echo time 28 ms, flip angle 70°, field of view 224×224×137 mm, 39 slices without slice gap, voxel size 3.5×3.6×3.5 mm. Two runs of 485 volumes each were obtained. MRI data acquisition took about 40 minutes.

### Data Processing and Statistical Analysis of fMRI

Preprocessing and statistical analyses of images were conducted with Statistical Parametric Mapping [43] version 8 (2008, Wellcome Department of Cognitive Neurology, London, UK; http://www.fil.ion.ucl.ac.uk/spm). All obtained volumes were used for data analysis. Preprocessing with SPM included realignment, coregistration and spatial normalization (template of Montreal neurological institute, MNI). Thereafter, an isotropic 8 mm full-with at half-maximum Gaussian filter was applied to smooth the data spatially.

All conditions were modelled in a block design for the statistical analyses of regional difference in cerebral activations. Conditions were implicitly contrasted at subject level against the rest conditions with a short central horizontal line. Regressors describing head motion linear, quadratic and derivative for the three linear and three rotational movement parameters were included.

Conditions were analyzed at group level by a three-way ANOVA for repeated measurements in which group, subject and condition were modelled. Group consisted of three levels, HC, PD_nFOG and PD_FOG. Condition consisted of five levels, FW, FtN, FtS, SW and StN. Subjects and groups were assumed to be independent, equal variant with regard to subject and unequal variant with regard to group. Conditions were assumed to be dependent and unequal variant due to different stimulus durations. As side of symptom onset has been suggested to influence the effect of optic flow in PD patients [24], this was included as covariate. Within and between groups comparisons were made. We compared wide-field flow (FW) with a static dot field (SW). The effects of interrupting wide-field flow (FtN and FtS) were both compared with narrowing a static dot field (StN). For FtN, this resulted in a well-balanced control condition. Although FtS and StN were not balanced for the dimensions of the visual field, StN controlled for effects due to change in the presented stimulus pattern. We additionally explored activation increases related to the three conditions characterized by a changing stimulus pattern (FtN, FtS and StN) when contrasted to the stable patterns (FW and SW). In this way, we aimed to gain insight in functional circuitry implicated in visual attention that might be relevant for understanding differences in visuomotor transformations between HC and PD.

The contrasts listed above were used for both within and between group comparisons. Thresholds for within group comparisons were initially set at *p*<0.001 (voxel-level uncorrected) and extent threshold (k) of 8 voxels. Comparing condition-related activations between groups was performed by explicit masking at *p* = 0.05 with a subsequent threshold *p*<0.001 (uncorrected, extent threshold 8 voxels). For each contrast, clusters corrected for whole brain volume at *p*<0.05 (FWE) were considered statistically significant. Cerebral activations were rendered on either T1 brain slices or on the surface of a standard MNI brain in SPM. Brain regions were identified by checking the coordinates onto the automated anatomical labelling template in MRIcron [44].

To explore differences between PD patients with either right or left dominant symptoms (PDR; PDL), we designed a second three-way ANOVA for repeated measurements in which again group, subject and condition were modelled. In this model, group consisted of the three levels HC, PDR and PDL. The presence or absence of FOG was included as covariate. Other settings equalled our first model. Within group comparisons and between group comparisons were set at the same thresholds.

### Functional Connectivity of Right V5

As the motion sensitive area V5 had the expected critical role in the current study [45], [46], and the right hemisphere has been described to play a dominant role in both normal visuomotor function [47], [48] and affected gait in PD [14], [16], we employed a functional connectivity analysis of right V5. Functional connectivity was assessed by using the psychophysiological interaction (PPI) method [49], which expresses the influence of neural activity of one over other cerebral regions. A nine mm sphere was selected around right V5 (x 48, y −70, z 0).

An individual PPI analysis was performed for each subject. PPI was computed as the cross-product of the extracted eigenvariate time series of right V5 and the convolved ‘psychological’ task effect (FW compared to SW). This PPI was entered as regressor in the first level PPI analysis along with two other regressors. The original eigenvariate of right V5 was included next to the convolved psychological variable, which results in a PPI analysis excluding the main effect of task but looks at the synchrony of neural activity between right V5 and other cerebral regions depending on the psychological variable (FW versus SW).

Brain regions with a positive regression slope with right V5 dependent upon the ‘psychological’ variable were identified for each subject. These contrasts were entered in a second-level ANOVA with three groups (HC, PD_nFOG and PD_FOG), which were assumed to be independent but unequal variant. The functional connectivity of right V5 depending on the wide-field flow versus the stationary wide field within a group were tested at *p*<0.05 voxel level corrected for whole brain volume (FWE). Between group differences were shown by exclusive masking using an initial threshold of *p*<0.05 (uncorrected) with a subsequent conservative threshold of *p*<0.05 voxel level corrected for whole brain volume (FWE). A cluster extend threshold of eight voxels was used for within and between group analysis.

### Correlation between Medial Premotor Activation and WAIS Block Design Test

The behaviorally assessed WAIS block design task for visuomotor abilities was expected to correlate with the ability to activate medial premotor areas during narrowing specifically the optic flow field (FtN>StN). To that end, we used the human motor area template [50] to create an independent region of interest constituted by the supplementary motor area (SMA) and pre-SMA, bilaterally. Extracting mean betas for this region was done using the MarsBar toolbox in SPM (http://marsbar.sourceforege.net). Mean beta’s during StN were subtracted from those obtained in FtN.

As we specifically expected that poorer visuomotor abilities measured by the WAIS block-design task resulted in reduced medial premotor activation, correlations with one sided *p*-values were used. A Pearson’s correlation was used for an uncontrolled correlation of the WAIS block design T-values (age corrected) with the medial premotor activations, while a partial correlation was used for calculating the correlation further controlled for block handling speed, MMSE, education level, UPDRS and PD onset-side.

## Results

### Group Characteristics

Basic characteristics of HC, PD_nFOG and PD_FOG did not significantly differ with regard to age (*F*(2,36) = 0.073, *p* = 0.930), education level (*H*(2) = 1.670, *p* = 0.434) and MMSE (*H*(2) = 2.828, *p* = 0.367 (Table 1). Neither were such differences found for block handling speed (*F*(2,36) = 2.492, *p* = 0.098) or performance on the WAIS block design task (*F*(2,36) = 1.954, *p* = 0.098). Significant differences were not seen either when the group of HC was compared with a single composite group of all PD patients.

Compared to PD_nFOG, PD_FOG patients had higher scores on the motor part of the UPDRS (*t*(20) = −3.696, *p* = 0.001) and rated higher on the FOG scale (*t*(20) = −6.202, *p* = 0.001). The latter included two questions concerning the impact of deteriorated gait, unrelated to the presence of possible FOG. No significant differences were seen between the two PD groups for the modified Hoehn and Yahr stage (*U* = 41.00, *p* = 0.367), PD duration (*t*(20) = −1.864, *p* = 0.77) and levodopa equivalent dose (*t*(20) = −1.619, *p* = 0.121) (Table 1). Overall, our PD patients were mild to moderately affected although advanced PD was not an exclusion criterion. On the other hand, patients with cognitive impairment (assessed with the MMSE) and patients who were considered unable to sustain the demand of the fMRI experiment were excluded. As such conditions are often associated with advanced PD, this may have led to an indirect exclusion of severely affected patients.

### Perceived Illusion of Forward Self-motion

Wide-field flow evoked a clear perception of forward self-motion in all three groups. This was underscored by the significant within group differences for the wide- and narrow-field presentations in flow and static mode (FW, FN, SW and SN; *p*<0.001). In FW, this illusion was stronger than in SW and SN for all groups. When compared to FN, the illusion in FW was rated significantly stronger (*p*<0.05) by HC and PD_nFOG, while PD_FOG did not perceive significant differences in forward self-motion between FW and FN (*p* = 0.231). This might be due to some residual flow in FN and the small number of PD_FOG patients. Comparing ratings between groups showed that the perception of forward self-motion was equally strong during FW for all groups; HC median 3 (interquartile range 1–7), PD_nFOG 4.5 (0–7), PD_FOG 5.5 (2–8); *H*(2) = 0.675, *p* = 0.713. In FN, this sensation was virtually absent for all groups: HC 0 (0–1.5), PD_nFOG 0 (0–0), PD_FOG 0 (0–5); *H*(2) = 2.263, *p* = 0.270. It was fully absent in SW and SN for all three groups (each 0; 0–0).

### Cerebral Activations Related to Wide-field Flow

**Figure 1:**
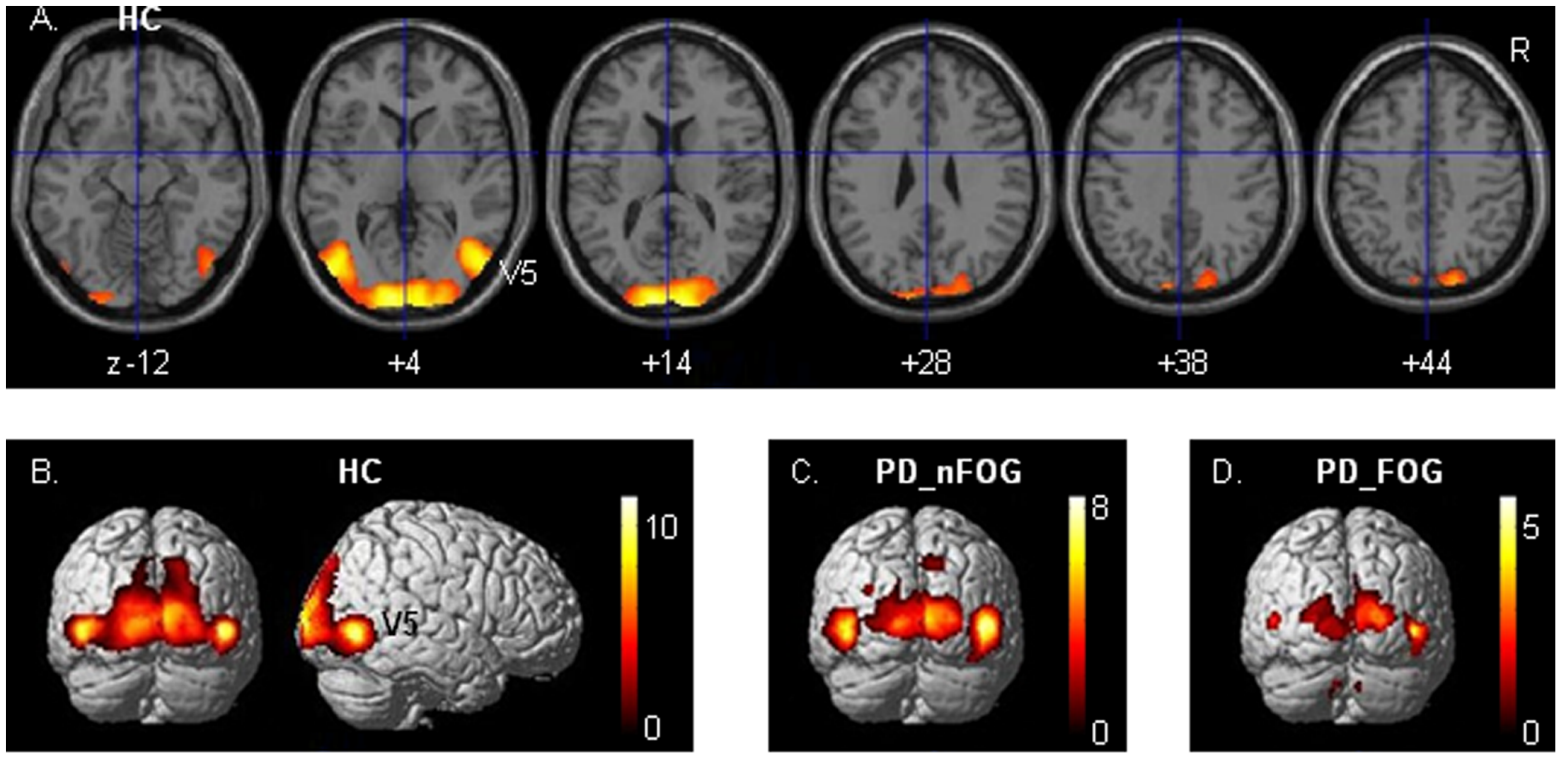
Wide-field optic flow. Cerebral activations related to wide-field radial optic flow (FW) contrasted to a wide stationary dot field (SW) in healthy controls (HC) are projected on transversal sections (A) and rendered onto brain surfaces (B). Activations in Parkinson patients without freezing of gait (PD_nFOG) and with freezing of gait (PD_FOG) are displayed in C and D, respectively. Thresholds are set at *p* = 0.001 voxel-level uncorrected with an extended voxel threshold of eight voxels. T-values are displayed in the color bars. R = right side of the brain, V5 = visual motion complex V5.

Cerebral activations related to FW (contrasted to SW), which evoked the illusionary perception of forward self-motion, were bilaterally distributed over striate and extrastriate visual cortical regions, including the putative visual motion area V5 (Fig. 1, Table 2). Particularly in HC, this pattern of activation extended dorsally into the posterior superior parietal cortex, with a significant cluster in the right-hemisphere (Table 2). Left-sided V1/V2 activation was significantly increased in HC relative to PD_nFOG, as revealed by exclusive masking of the HC pattern by the PD_nFOG activations (*p*<0.05, cluster-level corrected for brain volume; Table 2). Relative to PD_FOG, HC activation was increased in V1/V2 and V5, bilaterally, and in the right posterior parietal cortex. These activation differences (particularly in V5) similarly emerged from the pattern of activation increases in PD_nFOG when exclusively masked by the PD_FOG pattern. The FW-related right posterior parietal activation in HC, which was not seen in the two patient groups, was indeed significantly increased relative to only PD_FOG (exclusive masking; *p*<0.05 cluster-level corrected). Relative to PD_nFOG, such increase in HC only reached a subthreshold level of *p*<0.001 (voxel-level uncorrected).

**Table 2:** Cerebral activation in wide field flow contrasted to a wide stationary field.

Brain region	Left					Right				
	x,	y,	z	T-value	Extent	x,	y,	z	T-value	Extent
**HC**										
Cuneus V1/V2	−8,	−100,	12	9.33	2200	0,	−100,	0	10.05	sc
						24,	−96,	2	6.61	sc
V5 visual complex	−48,	−76,	0	9.49	545	52,	−72,	−2	11.40	633
	−42,	−90,	−4	5.00	sc					
Post. sup. parietal cortex						18,	−86,	46	5.55	sc
**PD_nFOG**										
Cuneus V1/V2	−10,	−96,	4	6.53	768	8,	−100,	10	7.62	sc
						22,	−92,	8	5.79	sc
V5 visual complex	−48,	−78,	2	8.18	405	50,	−72,	2	8.39	616
**PD_FOG**										
Dorsal occipital cortex						14,	−102,	14	5.75	65
V5 visual complex						46,	−72,	0	5.31	24
**HC excl PD_nFOG**										
Dorsal occipital cortex	−12,	−92,	18	6.60	10					
	−20,	−98,	−6	5.64	22					
**HC excl PD_FOG**										
Cuneus V1/V2	−12,	−92,	4	7.51	171	2,	−90,	6	5.87	sc
Dorsal occipital cortex	−4,	−94,	22	6.34	sc					
V5 visual complex	−48,	−68,	−2	7.41	176	50,	−62,	−2	6.62	46
Post. sup. parietal cortex						18,	−86,	44	5.53	12
**PD_nFOG excl PD_FOG**										
Cuneus V1/V2	−10,	−92,	4	5.48	10					
V5 visual complex	−48,	−80,	−4	8.07	105	54,	−76,	8	6.73	49

Regional activations related to FW contrasted to SW. Initial threshold was *p*<0.001 (uncorrected, extent threshold k = 8 voxels). The resulting single occipito-parietal cluster was constituted by distinct confluent regional activations. The latter were separated by thresholding at voxel level *p*<0.05, FWE-corrected. As none of the initially identified local maxima disappeared, the segregated clusters (*p*<0.05 cluster-level corrected for whole brain) are reported here. The reported co-ordinates represent maxima that are located more than 12 mm apart from the adjoining focus in each cluster. Positive x, y, z coordinates (in mm) indicate locations respectively right, anterior and superior to the middle of the anterior commissure. Abbreviations: HC = healthy controls; PD_nFOG = Parkinson patients without freezing of gait; PD_FOG = Parkinson patients with freezing of gait; excl = exclusive; post. = posterior; post. sup. = posterior superior; sc = same cluster; sup. = superior.

### Cerebral Activations Related to Wide-field Flow Interruption

**Figure 2:**
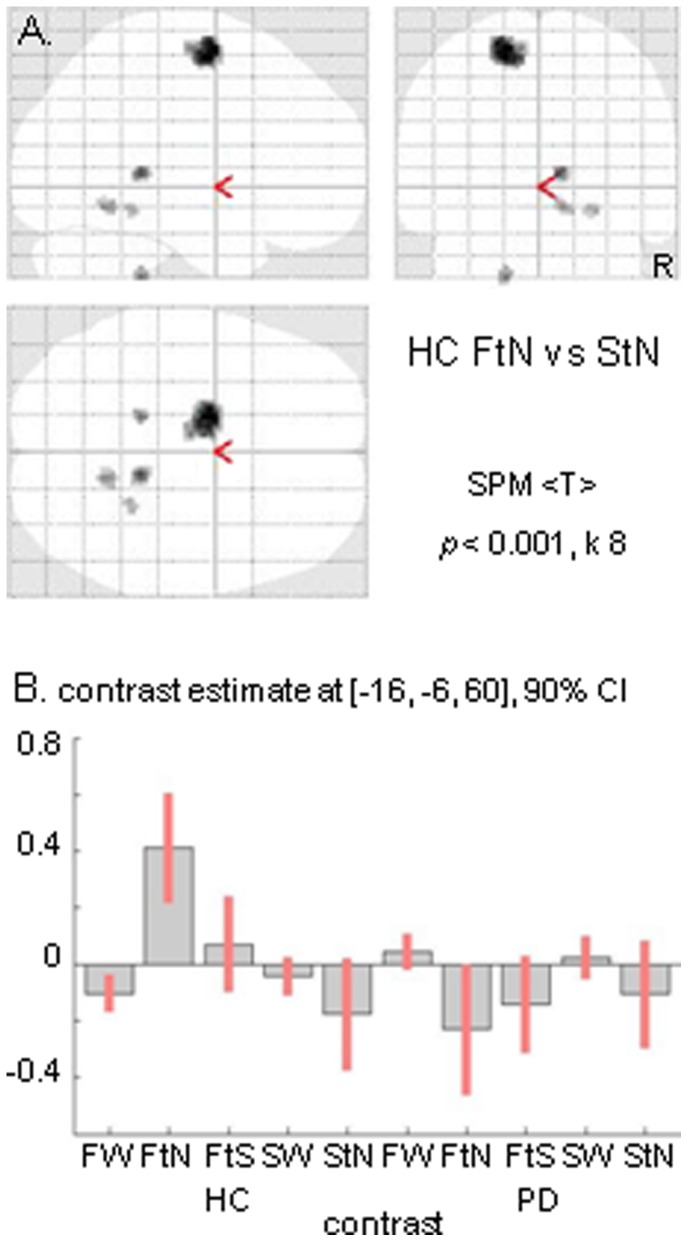
Narrowing wide-field optic flow. Cerebral activation related to the transition from wide to narrow optic flow (FtN) contrasted to narrowing of a stationary dot field (StN) in healthy controls (HC) is displayed in a glass brain view (A). Responses of the single significant cluster (max x −16, y −6, z 60) are shown for HC and the entire Parkinson’s disease (PD) group (B). Threshold is set at *p* = 0.001 uncorrected with an extended voxel threshold (k) of eight voxels. CI = confidence interval; FW = wide optic flow; FtS = gradual transition to stop wide optic flow; SW = wide stationary dot field. R = right side of the brain.

The crucial condition of gradually narrowing the wide-field flow (FtN) was expected to evoke stronger medial frontal activation in HC than in PD. Contrasting FtN to StN indeed resulted in a single cluster of significant activation in the SMA of HC, which extended in the pre-SMA (max. x −16, y −6, z 60; T-value = 4.95, *p*<0.05, cluster-level corrected) (Fig. 2). Such activation did not occur in either PD group. Exclusive masking of HC activation by PD_nFOG subtreshold results further demonstrated this increased (pre-)SMA activation in HC (*p*<0.05, cluster-corrected), which was similarly seen by exclusive masking of HC with PD_FOG. Applying such masking, no significant activation differences were seen between PD_FOG and PD_nFOG in FtN contrasted to StN. A trend of more (pre-)SMA impairment in PD_FOG than in PD_nFOG might be suggested by the lower contrast estimate for PD_FOG (−0.37) than for PD_nFOG (−0.10) during FtN, while during StN these values were −0.12 and −0.09, respectively.

The second condition of interrupting FW, i.e. by gradual deceleration of the radial dot flow (FtS), yielded a similar mediofrontal effect as FtN. Contrasting FtS to StN showed a cluster of increased activation (exclusive masking) in the (pre-)SMA of HC (max. x −2, y −2, z 66; T = 5.23), relative to PD_FOG as well as to PD_nFOG (each *p*<0.05, cluster corrected). Similarly, exclusive masking revealed increased (pre-)SMA activation in PD_nFOG relative to PD_FOG (*p*<0.05, cluster corrected). For the assessment of FtS, the same control condition StN was used as in the comparison with FtN. Although FtS and StN were not balanced for the spatial dimensions of the visual field, a control condition for non-specific change in the visual field appeared to be important (see results related to changing visual patterns). In addition to the FtS-related medial frontal activation, contrasting FtS to StN also resulted in bilateral activation in V1/V2 (only significant for the left hemisphere at *p*<0.05 whole-brain cluster corrected; max x 14, y −102, z 2), without significant differences between groups.

The reduced (pre-)SMA activation in PD at interrupting FW was hypothesized to mimic the impaired ability to internally recruit motor circuitry when external support falls away. We therefore assessed whether the magnitude of mediofrontal activation would be associated with a behavioral parameter concerning visuomotor control in PD. To that end, a bilateral SMA and pre-SMA template was applied to demarcate a volume of interest. Increased activation in this volume correlated with higher (age-corrected) scores on the WAIS block-design task in PD_nFOG patients (*r* = 0.54, *p* = 0.010). Although a similar trend was seen for PD_FOG patients as well as HC, correlations did not reach statistical significance (PD_FOG *r* = 0.29, *p* = 0.264; HC *r* = 0.36, *p* = 0.094). The correlation in PD_nFOG became even stronger when further controlled for block handling speed, MMSE, education level, UPDRS and onset-side (*r* = 0.80, *p* = 0.003).

As the involvement of a ‘mesencephalic locomotor region’ has recently been described to be either hyperactive [51] or impaired in FOG [52], we looked for subthreshold effects at the described location (*p*<0.05, voxel-level uncorrected). FtN contrasted to StN showed a small cluster (9 voxels) in HC (x 6, y 28, z −20; T-value = 2.07), which was not seen in either PD_FOG or PD_nFOG. Although our observation does not concern a significant activation, it would best fit with the results of Shine and colleagues [52].

### Effect of Side of PD Symptoms on Wide-field Flow Activation

**Figure 3:**
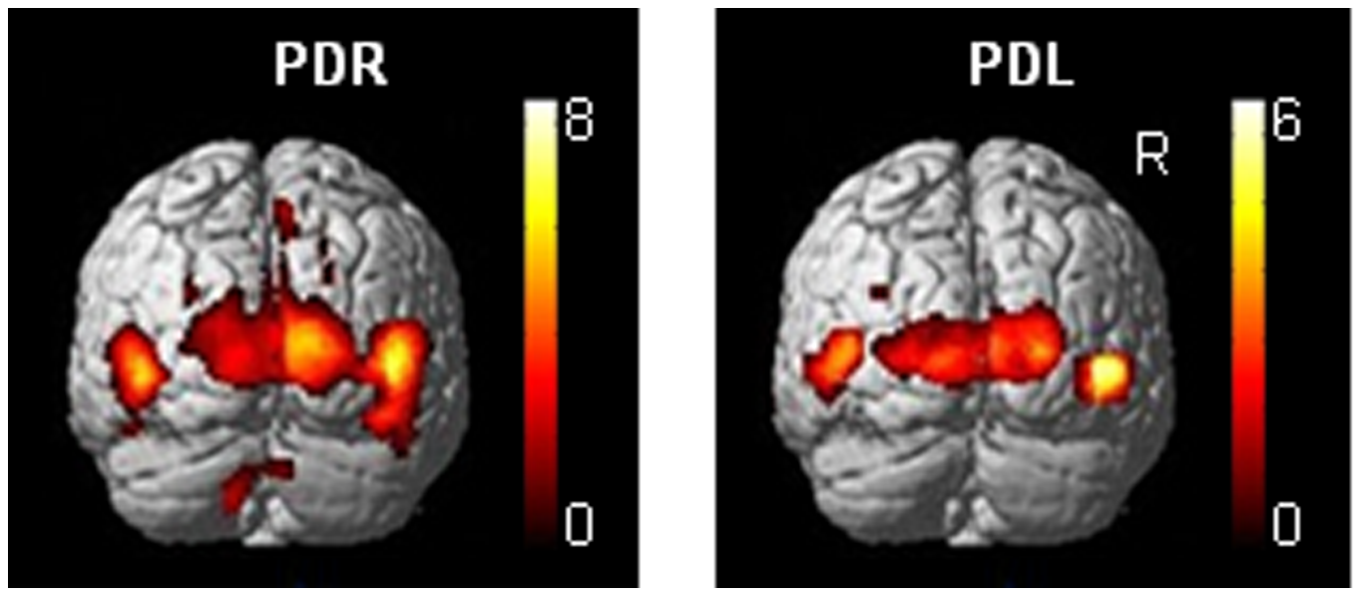
Wide-field optic flow and lateralization of symptoms. Cerebral activations related to wide-field flow (FW) contrasted to a wide stationary field (SW) are rendered onto a brain surface (posterior view) for thirteen right symptom dominant Parkinson patients (PDR) and nine left symptom dominant Parkinson patients (PDL). Thresholds are set at *p* = 0.001 uncorrected with an extended voxel threshold of eight voxels. T-values are displayed in the color bars. R = right side of the brain.

Given a general dominance of the right hemisphere in visuospatial processing, we explored possible activation differences between hemispheres related to the side of symptom dominance in PD patients. To that end, PD patients with right and left dominant symptoms (PDR, *N* = 13 of which 5 FOG; PDL, *N* = 9 of which 2 FOG) were compared with a separate ANOVA controlling for FOG binary. The only difference in brain activation was seen in FW contrasted to SW (Fig. 3, see further). No significant differences were seen between PDR and PDL during the interruption of FW, neither in FtN nor in FtS. At subthreshold level (*p*<0.05, cluster-level, uncorrected) activation differences were not seen either.

Relative to PDR, the PDL patients showed decreased activation during FW contrasted to SW (*p*<0.05, cluster corrected; PDR increases exclusively masked by PDL) in the dorsal extension of right V5 (x 40, y −66, z 8) and a right lateral occipital area at a more ventral location (x 46, y −70, z −16) (Fig. 3). A right posterior parietal activation decrease (x 8, y −78, z 54; T-value = 3.96) remained at subthreshold significance level, which was similarly the case for left V5 activation. No activation decreases were seen in PDR relative to PDL (using exclusive masking). PDR patients showed a similar distribution of activations as HC, while PDL patients showed similar changes relative to HC as to PDR.

### Cerebral Activation Related to Changing Stable Visual Patterns

**Figure 4:**
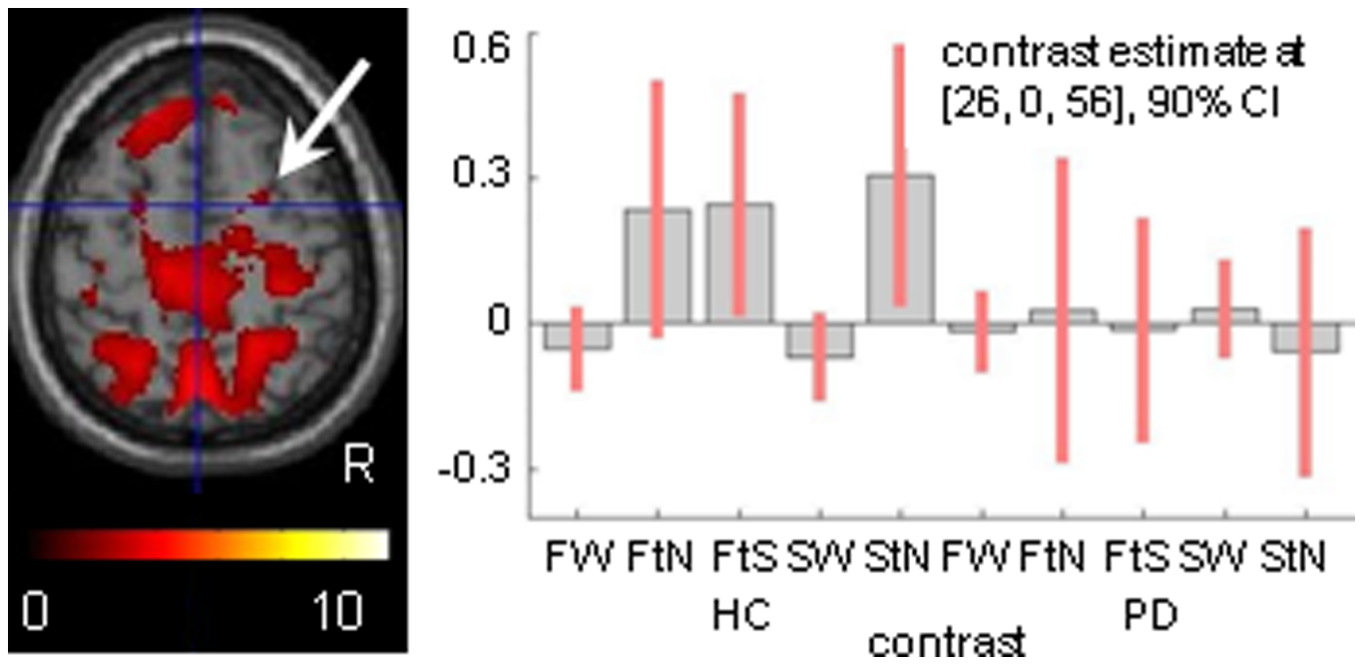
Right dorsal premotor responses in wide-field optic flow. Contrasting wide-field optic flow (FW) to a wide stationary field (SW) revealed a hardly detectable FW-related effect in the right dorsal premotor cortex of healthy controls (HC) by a threshold of *p* = 0.5 (extended voxel threshold eight voxels). However, the response pattern at this location (max. x 26, y 0, z 56) was particularly characterized by increased activation related to the ‘change’ conditions in HC, compared to the stable conditions FW and SW. Moreover, these increases were not seen in the Parkinson patients (PD). Abbreviations are as in figure 2.

**Figure 5:**
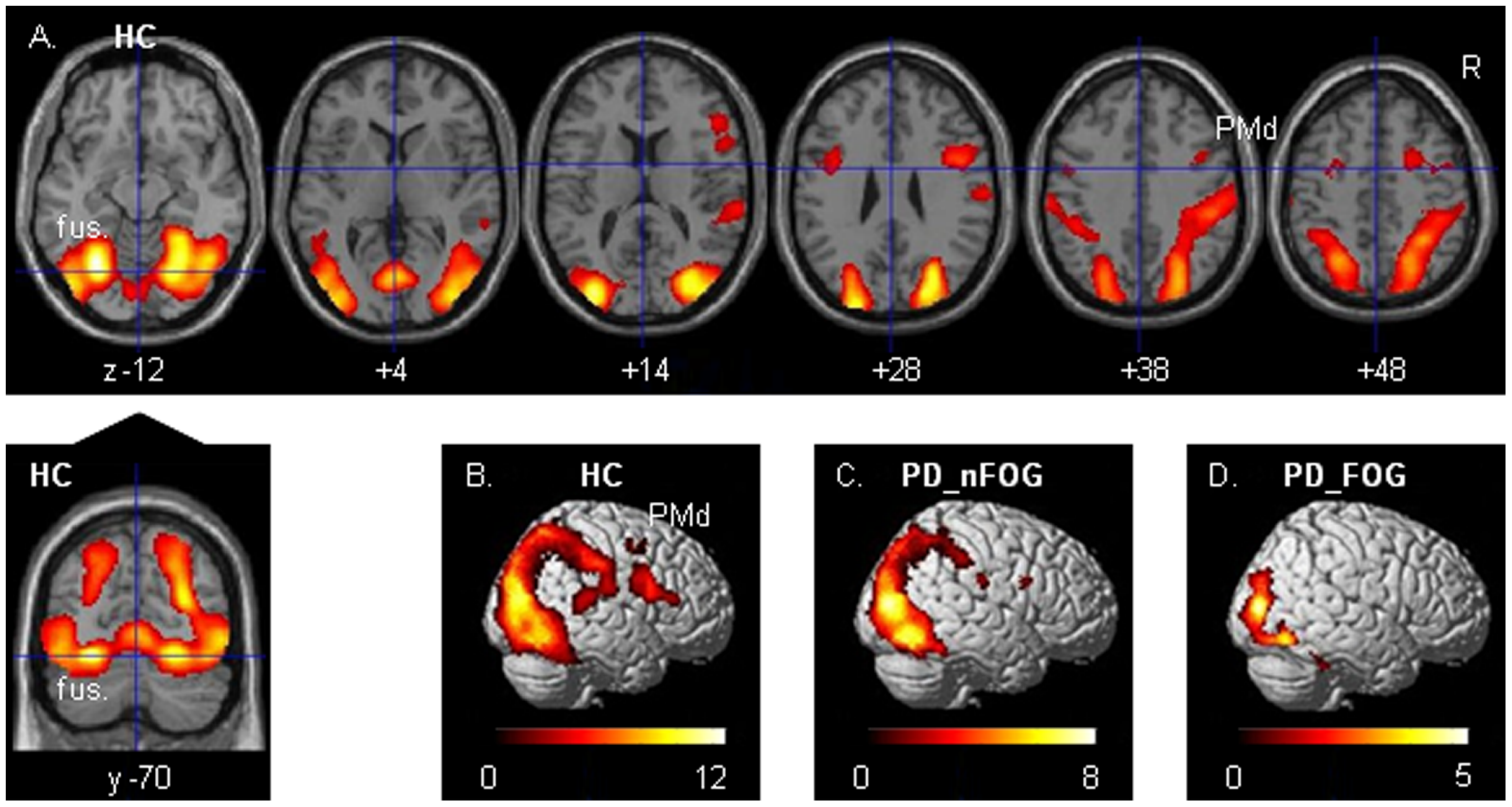
Non-specific ‘change’ in the stimulus pattern. Cerebral activations related to the conditions with non-specific ‘change’ in the presented visual stimuli (FtN, FtS, StN) contrasted to the stable stimulus patterns (FW, SW) in healthy subjects (HC) are projected on transversal sections and a coronal one (A) and rendered onto brain surfaces (right lateral view; B). Activations in Parkinson patients without freezing of gait (PD_nFOG) and with freezing of gait (PD_FOG) are displayed in C and D, respectively. Thresholds are set at *p* = 0.001 uncorrected with an extended voxel threshold of eight voxels. T-values are displayed in the color bars. Fus. = fusiform gyrus, PMd = dorsal premotor cortex. Other abbreviations are as in figure 2.

Two observations from the preceding results were a reason for additional analysis. First, in contrast to our expectation [35], we did not find (right) dorsal premotor (PMd) activation related to FW. Second, V5 activation was not restricted to FW, but appeared to be strong in both FtN and StN as well. As V5 activation was similarly strong in these two conditions, the specificity of particularly interrupting FW by FtN was adequately balanced by the introduction of StN, controlling for V5 activation due to the apparent motion effect of gradually narrowing the dot field. It did, however, point at a hidden variable. Plotting the effect size at the PMd site of hardly detectable change in FW contrasted to SW (x 26, y 0, z 56) revealed a characteristic increase of activations related to exclusively FtN, FtS and StN in HC only (Fig. 4). As this profile of responses distinguished the conditions with change in the visual stimulus pattern, we subsequently contrasted these three conditions of change to the stable patterns FW and SW. This resulted in a robust pattern of significant activation increases in HC comprising fusiform gyri, putative V5, the anterior portion of the inferior wall of the calcarine sulcus (peripheral field representation of V1), dorsolateral extrastriate visual cortex, medio-posterior (superior) and antero-lateral (inferior) parietal cortex, bilaterally, right PMd and the dorsolateral prefrontal cortex of both hemispheres (Table 3, Fig. 5a–b).

**Table 3:** Cerebral activation in changing versus stable visual stimulus patterns.

Brain region	Left						Right					
	x,	y,	z	T-value	Extent		x,	y,	z	T-value	Extent	
**HC**												
Fusiform gyrus	−28,	−66,	−10	12.67	22809		30,	−52,	−16	11.21	sc	
							24,	−70,	−10	10.33	sc	
V5 visual complex	−46,	−78,	−10	9.08	sc		48,	−62,	−12	8.85	sc	
							42,	−84,	−2	9.39	sc	
Lingual gyrus (V1/V2)							6,	−74,	−2	9.64	sc	
Middle temporal gyrus							34,	−32,	2	5.21	sc	
							48,	−32,	8	6.43	sc	
Dorsolat. occipital cortex	−32,	−86,	16	11.35	sc		28,	−82,	20	11.50	sc	
Post. sup. parietal cortex	−24,	−78,	40	6.44	sc		26,	−62,	60	7.41	sc	
	−26,	−52,	52	8.60	sc							
Ant. inf. parietal cortex	−34,	−46,	46	4.90	sc							
Dorsolat. prefrontal cortex	−38,	4,	28	4.30	333		48,	4,	28	5.89	1097	
	−50,	−2,	42	3.52	sc		56,	18,	18	4.33	sc	
Dorsal premotor cortex							24,	2,	50	4.21	238	
							44,	−2,	54	3.55	sc	
**PD_nFOG**												
Fusiform gyrus	−22,	−74,	−10	6.57	14135		28,	−62,	−10	8.05	sc	
V5 visual complex	−48,	−78,	−10	6.75	sc		44,	−72,	−12	7.26	sc	
Lingual gyrus (V1/V2)	−4,	−76,	2	6.97	sc							
Dorsolat. occipital cortex	−50,	−80,	6	5.81	sc		40,	−84,	10	7.95	sc	
	−28,	−88,	18	7.53	sc		26,	−80,	28	7.48	sc	
	−28,	−74,	28	4.85	sc							
Post. sup. parietal cortex	−18,	−74,	46	5.99	sc		24,	−68,	34	6.67	sc	
	−26,	−54,	48	5.49	sc		30,	−54,	50	5.73	sc	
**PD_FOG**												
Lateral fusiform gyrus	−44,	−64,	−16	3.54	884		40,	−80,	−14	4.48	1410	
							44,	−68,	−14	5.09	sc	
V5 visual complex	−48,	−80,	−10	4.36	sc							
Lingual gyrus (V1/V2)							6,	−76,	−2	4.70	253	
Dorsolat. occipital cortex	−24,	−84,	14	5.34	sc		36,	−88,	8	5.12	sc	

Regional activations related to [FtN, FtS, StN] contrasted to [FW, SW], initial voxel-height threshold *p*<0.001 (uncorrected, extent threshold k = 8 voxels). Coordinates refer to the voxels of maximum activation within clusters of significant activation (*p*<0.05, FWE whole brain cluster-level corrected). The presented local maxima are located more than 12 mm apart from the adjoining focus in a cluster. Positive x, y, z coordinates (in mm) indicate locations respectively right, anterior and superior to the middle of the anterior commissure. Abbreviation: HC = healthy controls; PD_nFOG = Parkinson patients without freezing of gait; PD_FOG = Parkinson patients with freezing of gait; dorsolat. = dorsolateral; post. sup. = posterior superior; sc = same cluster.

**Figure 6:**
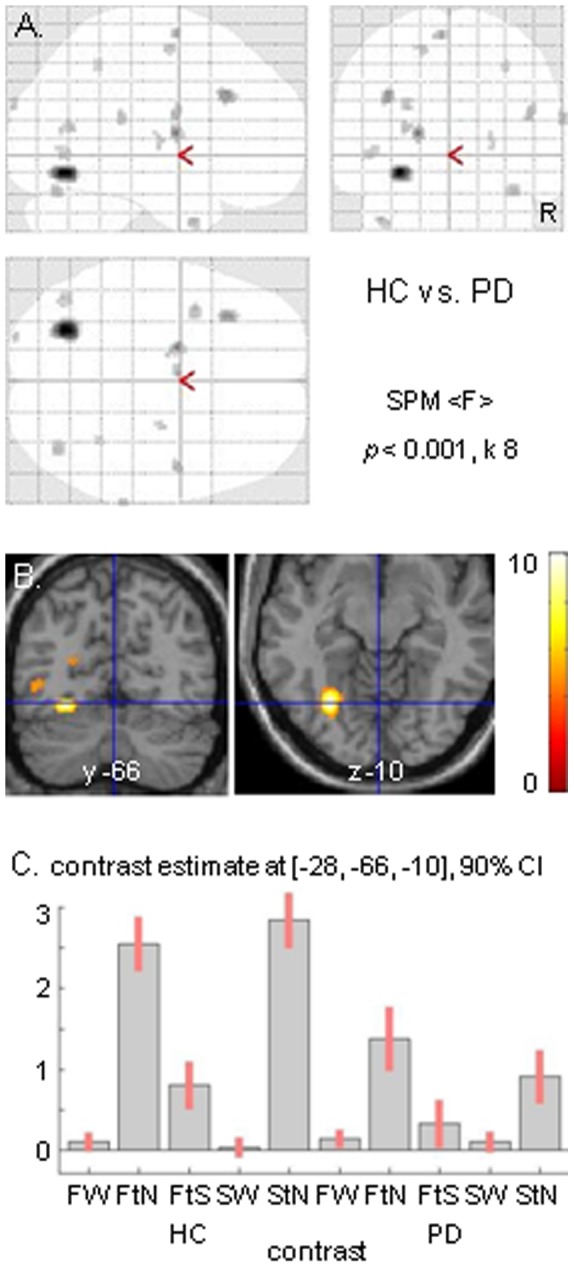
Main difference between healthy controls and Parkinson patients. The F contrast of the differences between healthy controls (HC) and the entire Parkinson group (PD) highlights the left fusiform gyrus, shown in a glass brain view (A) and by sections in both transversal and coronal sections (B). Responses of the left fusiform gyrus in the experimental conditions are shown for HC and PD patients (C). Conventions and abbreviations are as in figure 2.

In PD_nFOG, these activations related to visual change were less robust, while PMd activation was absent (Table 3, Fig. 5c). PD_FOG showed even less activation, without medial fusiform and parietal activations (Table 3). The importance of left fusiform function in our paradigm was further underscored by the fact that this focus of activation (x −28, y −66, z −10) was the main denominator that discriminated the HC and PD groups (F-test HC contrasted to PD; *F* = 10.02, *p* = 0.002, FWE-corrected at voxel level) (Fig. 6). We did not find a correlation of this fusiform gyrus activation with either UPDRS or Hoehn and Yahr scores in the PD patients. Neither was a correlation seen with the WAIS block design task or illusion of FW-related forward self-motion in either HC or the PD groups.

### Distant Effects of Right V5

**Figure 7:**
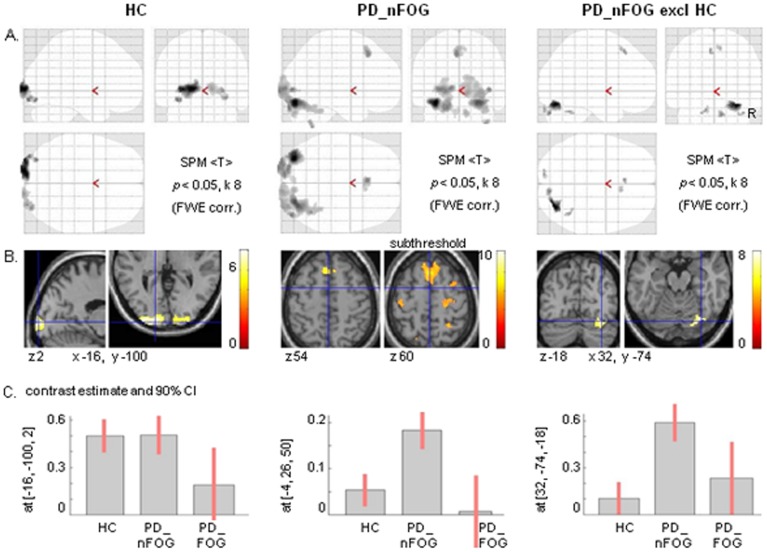
Functional connectivity of right V5. Cerebral regions that were identified by their functional connectivity with the right visual motion area V5 (using psychophysiological interaction, PPI) during wide-field radial optic flow (FW) contrasted to a wide stationary field (SW). These regions are displayed in glass brains (A) and projected on sections (B) for healthy controls (HC), Parkinson patients without freezing of gait (PD_nFOG) and for the latter when exclusively masked with HC at *p* = 0.05. Thresholds are set at *p* = 0.05 FWE corrected for whole brain volume with an extended voxel threshold of eight voxels. In addition, subthreshold effects in a transversal section at z = 60 are shown for PD_nFOG, threshold T = 4.5 (extended cluster threshold of eight voxels). T-values are displayed in the color bars. The corresponding graphs (C) show the contrast estimates and 90 percent confidence intervals (CI) for the maxima in the left fusiform gyrus, the pre-SMA and right cerebellum respectively.

As FW contrasted to SW revealed a general reduction of visual cortex activation in PD and no associated premotor activation, we tested whether particularly the right visual motion area V5 (x 48, y −70, z 0) might nevertheless have a stronger influence on premotor regions in PD than in HC. Functional connectivity using PPI revealed significant effects in V1/V2 of HC (Fig. 7) while in PD_nFOG additional effects were particularly seen in the medial prefrontal cortex, pre-SMA (x −4, y 26, z 50, dorsally extending onto plane z 58), and the anterior lobes of the cerebellum (*p*<0.05, FWE cluster corrected) (Fig. 7). No significant distant effects were seen from right V5 in PD_FOG. Exclusive masking of the effects in PD_nFOG by the HC effects showed that the V5 effects on the medial prefrontal cortex (left dominant) and right cerebellum were stronger in this patient group than in HC (Fig. 7).

## Discussion

Wide-field radial optic flow, which generated the illusionary perception of forward self-motion in both HC and PD patients, was related with reduced visual cortex activation in PD patients. On the other hand, functional connectivity of right visual motion area V5 with medial prefrontal cortex (pre-SMA) and cerebellum was stronger in PD_nFOG than in HC. The latter might point at functional circuitry implicated in enhanced stimulus effects on movement (including locomotion) known to occur in PD. The conditions with gradual transition from wide-field flow to either narrow flow or a wide static field, which was considered to mimic a circumstance requiring a stronger internal effort to maintain (virtual) locomotion when visual support falls away, evoked (pre-)SMA activation only in HC and not in the two PD patient groups. The number of included patients in the PD_FOG group (*N* = 7) was small, which is a reason to refrain from drawing firm conclusions concerning this PD subgroup. Keeping this restriction in mind, the obtained patterns of activation generally suggest that PD_FOG represented advanced PD relative to PD_nFOG (*N* = 15), which also emerged from the UPDRS differences. Finally, we identified a distribution of activations related to non-specific ‘change’ in the presented patterns of stimuli. In HC, these activations included the dorsal occipital-parietal cortex lateral to the regions of wide-flow induced activation, together with fusiform gyrus and right premotor regions. These activations were less robust in PD, suggesting PD-related changes in visuospatial attention.

### Reduced Visual Cortex Activation and Enhanced Distant Interactions in PD in Wide-field Flow

Wide-field optic flow generated the illusionary perception of forward self-motion in all three groups. In HC, the flow-related activations comprised the visual motion area V5, ventrally extending on the right side, posterior V1/V2 and dorsal extrastriate visual cortex extending more strongly in the right parietal cortex. This dorsal pathway activation, representing circuitry implicated in dynamic visuospatial processing [53], [54], was less robust in PD. Given the behavioral observations of stronger stimulus effects on motor control in PD than in HC, the reduction of PD visual cortex activation and the absence of dorsal premotor involvement in FW (contrasted to SW) might, at first sight, suggest the opposite effect, i.e. a reduced information flow onto motor-related circuitry. On the other hand, reduced visual activation may reflect a less elaborate level of information processing within the visual cortex. Such visual information is processed in a highly segregated fashion, with parvo (color) and magnocellular (motion) streams that can be distinguished at the retinal level and in the striate and extrastriate visual cortex [55]. In PD, these basic visual processing streams may be affected independently [56]. However, visual cortex processing is not only characterized by such segregation as integration plays an important role too [57]. Feedback is one of such integration processes, which is reflected e.g. in the variance whether visual motion input in V1 precedes or follows input in V5 [58]–[60]. Reduced visual activations and enhanced functional connectivity of right V5 with medial frontal-cerebellar circuitry in PD might thus reflect impaired visual cortex integration, with the consequence of dominating distant interactions along the segregated processing stream of visual motion funnelled through V5.

Enhanced functional connectivity between visual processing sites and distant action-related regions, with a reduced contribution of intermediate occipito-parietal regions, might result in less fine-tuned responses to visual stimuli, i.e. inducing responses that are either too strong or too weak. Such unbalanced responses due to ‘by-passing’ optimal parietal processing in sensori-motor planning would be consistent with the role of the parietal cortex providing ‘sensory’ information for feedforward processing in motor planning [61]–[64]. This means that in normal conditions, the predicted sensory consequences of planned movements are used to optimize preparation of such movements. Such parietal feedforward function is supported by interactions with the cerebellum [65]–[69]. The association of medial prefrontal (pre-SMA) and cerebellar functions, inferred from the increased functional connectivity of right V5, might point at an alternative, compensatory strategy for impaired sensation-based feedforward processing in PD. The role of the (pre-)SMA in such compensation would fit its involvement in error detection and serial ordering [70]–[72]. In this, compensation would be based on a mechanism of error correction facilitated by a ‘wider reference frame’ for assessing predictions. Wider referencing implies that sensory predictions are not restricted to immediate movement fragments but are made in the context of a larger time scale with movement patterns constituting series of events [73]–[76].

### Interrupting Wide-field Optic Flow and Activation of the (pre-)SMA

Narrowing the wide flow field evoked a single significant cluster of (pre-)SMA activation in HC and not in the two PD groups (FtN contrasted to StN). This confirmed the main hypothesis of our study. A lower contrast estimate in PD_FOG than in PD_nFOG might hint at more (pre-)SMA impairment in PD_FOG. Interrupting wide-field flow by gradual deceleration (FtS contrasted to StN) did also reveal medial frontal activation comprising SMA and pre-SMA in HC, while at this focus of maximum activation, a strong reduction was seen in PD_FOG relative to PD_nFOG. It should be recognized that the activation increase resulting from FtN contrasted to StN was most specifically associated with the interruption of perceived forward self-motion because possible effects of e.g. attention to change, due to narrowing itself, was balanced by narrowing a static dot field (StN). The other condition of interruption (deceleration, FtS) was controlled for non-specific change by similarly using StN, although FtS and StN were not optimally balanced for visual field dimensions. In both FtN and FtS, recruitment of the (pre-)SMA is considered to reflect its contribution to an internal drive to continue virtual locomotion when external support from visual flow falls away. This is consistent with the crucial involvement of the SMA and pre-SMA in the internal generation of movements, relative to externally driven action [28], [77]. The fact that our PD patients were compromised in such SMA recruitment fits the previously described functional impairment of this important outflow target of the basal ganglia [30], [32], [78]–[80].

As phrased before, the small number of patients with FOG does not allow final conclusions concerning changes in circuitry related to this symptom. However, reduced activations and higher UPDRS motor scores suggest that PD_FOG concerns a more advanced stage of disease, although the Hoehn and Yahr range between 2 and 2.5 indicates that differences were subtle. Reduced (pre-)SMA activation in PD at the loss of (virtual) gait-support thus appears to be a more general than a specific indicator related to FOG, pointing at an important role in visuospatial coordination which fails at specific epochs of changing visual motion in PD. Previous fMRI activation studies addressing FOG in PD made use of visuomotor tasks that included either imagery walking in virtual reality [51] or actual bipedal movements providing visual feedback by virtual progression in a displayed corridor [52]. Snijders and co-workers found hyperactivity in the ‘mesencephalic motor region’ during gait imagery in PD_FOG (relative to PD_nFOG), while reduced activations were seen in medial frontal regions. In the study of Shine and co-workers, reduction of bipedal movements (virtual FOG) was induced by visual instructions that inflicted an increased cognitive load in PD patients. These changes resulted in lateral fronto-parietal activation increases and decreased activation of the sensorimotor cortex and caudate nucleus. Controlled for the visually presented cognitive load, region of interest analysis showed reduced activation in the ‘mesencephalic motor region’, which correlated with the FOG severity.

Foci of reduced medial frontal activations associated with PD in general as well as PD_FOG was a common finding in our study and that of Snijders and co-workers, including the variance in the condition-specific foci of maximum activation within SMA, pre-SMA and cingulate motor cortex. With regard to effects specifically associated with FOG, e.g. on the ‘mesencephalic motor region’, final conclusions cannot be drawn from our study due to the low number of PD_FOG patients. Moreover, one may assume that in the Snijders study, compared to a strict visual stimulus paradigm, motor imagery did recruit more extended motor-related circuitry, indeed enabling identification of such FOG-related change in mesencephalic activation which we did not. In this respect, these studies are complementary because our study highlighted the PD-associated functional changes in medial frontal and visual cortex regions when challenged by a specific visual motion stimulus. In addition, we demonstrated a distribution of activations associated with non-specific ‘change’ in the presented visual pattern that resembled previously described spatial attention networks [81]–[83]. The lateral fronto-parietal regions of activation decrease in our PD patients (relative to HC) were similar to the fronto-parietal activations associated with inhibited bipedal movements due to cognitive load in the PD patients of Shine et al. [52]. While in their study, an increased cognitive demand was intrinsically associated with both a decrease of bipedal movements and the consequent slowing of virtual progression in the displayed corridor, our analysis enabled a dissociation between global attention to spatial change and the specific effect of interrupting wide-field flow, i.e. interrupting the illusionary perception of forward self motion.

### Attention to Non-specific Spatial Change

The absence of right PMd activation was a reason to explore subthreshold responses in this cortical region, which revealed that responses at this location were specifically evoked by change of the stimulus pattern, i.e. FtN, FtS as well as StN. Moreover these responses were significantly stronger in HC than in PD. Contrasting the conditions characterized by ‘change’ to the ‘stable’ stimulus patterns FW and SW resulted in a distribution of robust activations bilaterally comprising the fusiform gyrus, dorsolateral visual, parietal and dorsolateral prefrontal cortex, together with right-dominant PMd activation. Particularly the dorsal parietal-PMd-prefrontal pattern of activations pointed at a functional network implicated in spatial attention processing [81]–[83]. Although subjects were not instructed to explicitly detect changes in the presented stimulus patterns, differences between the conditions made it plausible to infer that the ‘change’-related distribution of activations indeed represented a neuronal mechanism of processing covert spatial attention. In PD patients such mechanism seems to be impaired.

While goal-directed attention to distinct features in a visual scene involves top-down processing, covert attention points at bottom-up mechanisms of detecting unpredictable change [84]–[87]. The fusiform gyrus can be recruited in both ways. The circumstance of covert attention (to change) in our experiment provides the main argument to assume that its activation is particularly due to bottom-up processing, while fusiform gyrus participation in an otherwise dorsal visual pathway points at a visuospatial function associated with novelty detection or saliency processing [88], [89]. Classically, the fusiform gyrus plays a prominent role in the ventral visual stream concerning e.g. shape and object recognition, which is distinguished from dorsal pathway processing of visuospatial characteristics [90], [91]. On the other hand, early ventral occipito-temporal contributions to spatial processing are particularly revealed in experimental settings with objects placed outside a target point of central fixation [85], [92]–[94]. Functional coherence of the fusiform gyrus and parietal cortex, as we found in the ‘change’ conditions of our experiment, may therefore be associated with the assessment of ‘objects’ (or basic shapes) in a dynamic environment. Such assessment becomes relevant during e.g. locomotion, when unexpected (extra-fovealy projected) obstacles need to be avoided [23], [95], [96]. Changes in the visual patterns we employed indeed occurred in primarily the peripheral visual field. This held for narrowing the dot field, both in flow and static mode, as well as for deceleration, which implied that the largest change of radial dot speed occurred in the peripheral part of the display. An additional argument that ‘change’-related activations predominantly occurred in the peripheral visual field can be concluded from the anterior calcarine activation, which topologically represents the peripheral field [97], [98].

In addition to our proposal that impaired visual motion processing in PD leads to impaired motor preparation due to reduced feedforward processing, one may also consider that impaired novelty detection in a changing visually recorded environment may lead to delate motor adjustments. This seems consistent with the previously suggested disruption of (right-hemisphere) visual and ‘executive-attention’ networks in PD_FOG [99]. A consequence would be that in visual circumstances requiring stronger internally driven motor control, this may not only fail in PD due to impaired medial prefrontal function, but the initial cue for such recruitment may not be strong enough either. On the other hand, the specificity of our (pre-)SMA activation in HC at interrupting the illusion of forward self motion was due to contrasting two balanced ‘change’ conditions (FtN versus StN), thus pointing at a response strongly associated with higher-order network consequences of visual motion processing such as e.g. motor intention. The general ‘change’ activations included lateral but no medial prefrontal regions, neither in HC nor in PD. This underscores the distinction between neuronal circuitries related either to general visual attention or to specific visual motion processing in our paradigm. One might oppose that lateral fronto-parietal activations related to the ‘change’ conditions may similarly reflect top-down visual processing [87], [100]. We agree that the latter may play a role too and that in PD, impaired attention may contribute in various ways to dysfunction of motor behavior, including gait [101], [102]. The fact, however, that our design did not include an explicit attention condition, favors a dominance of bottom-up attention-related processing.

### Right Hemisphere Impairment in PD Visuomotor Control

The absence of a correlation between FOG and the WAIS block design task did not provide support for a relation between FOG and right-hemisphere dysfunction in PD, although such correlation has previously been reported [103]. The low number of PD_FOG patients may be due to this negative result. On the other hand, the correlation between (pre-)SMA activation during FtN and the WAIS block design task indicates that impaired right hemisphere function is associated with the reduced ability to recruit an internal drive to maintain virtual locomotion when the perceived forward self-motion is interrupted. This might support the discussed option that impaired bottom-up visual motion processing fails to provide the optimal cues for (pre-)SMA activation. More support for involvement of the right hemisphere came from differences between patients with right and left dominant symptoms during wide-field optic flow. In this condition, PDR patients (dominated by left hemisphere disease) showed more activation of the dorsal stream (including the right posterior parietal cortex) compared to PDL patient (dominated by right hemisphere disease). These results point at consistency with previously described right-hemisphere dominance concerning visuomotor transformations [12], [104], including gait [105] and optic flow-derived visuospatial perception in PD [16]. Furthermore, they fit well with a previously suggested relation between left symptomatic PD and freezing of gait occurrence [106].

## Conclusions

Our findings indicated that the compromised ability of PD patients to internally generate action for maintaining virtual locomotion when external support of wide-field flow falls away is based on impaired (pre-)SMA activation. Reduced dorsal occipito-parietal activation during wide-field flow in PD was argued to reflect reduced visuo-spatial integration, with the effect that predicted sensory consequences of movements cannot be optimally implemented in motor preparation. This impaired occipito-parietal function was associated with enhanced functional connectivity of a segregated magnocellular functional stream through right visual motion area V5 with distant medial fronto-cerebellar circuitry. In this way, compensation of impaired early-stage feedforward processing is logically based on a shift to more distant fronto-cerebellar feedforward processing. The latter implies, however, that motor responses to visual motion stimuli may be either too strong or too weak in PD. In addition, we identified a seperate pattern of activations related to non-specific stimulus change which pointed at covert spatial attention and impairment of such function in PD.
